# Non-conventional pathways enable pennycress (*Thlaspi arvense* L.) embryos to achieve high efficiency of oil biosynthesis

**DOI:** 10.1093/jxb/eraa060

**Published:** 2020-02-01

**Authors:** Enkhtuul Tsogtbaatar, Jean-Christophe Cocuron, Ana Paula Alonso

**Affiliations:** 1 Ohio State Biochemistry Program, The Ohio State University, Columbus, OH, USA; 2 BioDiscovery Institute, University of North Texas, Denton, TX, USA; 3 Department of Biological Sciences, University of North Texas, Denton, TX, USA; 4 MPI of Molecular Plant Physiology, Germany

**Keywords:** ^13^C-labeling, carbon conversion efficiency, isocitrate dehydrogenase, oilseed, pennycress, plant metabolism, Rubisco

## Abstract

Pennycress (*Thlaspi arvense* L.) accumulates oil up to 35% of the total seed biomass, and its overall fatty acid composition is suitable for aviation fuel. However, for this plant to become economically viable, its oil production needs to be improved. *In vivo* culture conditions that resemble the development of pennycress embryos *in planta* were developed based on the composition of the liquid endosperm. Then, substrate uptake rates and biomass accumulation were measured from cultured pennycress embryos, revealing a biosynthetic efficiency of 93%, which is one of the highest in comparison with other oilseeds to date. Additionally, the ratio of carbon in oil to CO_2_ indicated that non-conventional pathways are likely to be responsible for such a high carbon conversion efficiency. To identify the reactions enabling this phenomenon, parallel labeling experiments with ^13^C-labeled substrates were conducted in pennycress embryos. The main findings of these labeling experiments include: (i) the occurrence of the oxidative reactions of the pentose phosphate pathway in the cytosol; (ii) the reversibility of isocitrate dehydrogenase; (iii) the operation of the plastidic NADP-dependent malic enzyme; and (iv) the refixation of CO_2_ by Rubisco. These reactions are key providers of carbon and reductant for fatty acid synthesis and elongation.

## Teaser

Pennycress embryos are extremely efficient in converting substrates into biomass. Non-conventional pathways, such as the reversibility of isocitrate dehydrogenase, and CO_2_ refixation by Rubisco enable this high biosynthetic efficiency.

## Introduction

Pennycress (*Thlaspi arvense* L.), a member of *Brassicaceae* family, is a winter annual that grows in temperate regions of North America (Hojilla-Evangelista *et al.*, 2013). It germinates in the autumn, and forms a low-growing rosette as it overwinters. Following the flowering period in the spring, seeds are harvested in May before summer crops are planted (Fan *et al.*, 2013). Thus, pennycress has a potential to grow in a summer/winter rotational cycle with other conventional commodity crops such as corn and soybean. While pennycress grows in a field, it can also serve as a cover crop by utilizing excess nitrogen, slowing soil erosion, and suppressing weeds (Phippen and Phippen, 2012). Additional advantages of pennycress include tolerance of fallow lands and minimal agricultural inputs such as pesticides, fertilizer, and water (Moser *et al.*, 2009). Moreover, in recent years, pennycress has been recognized as an oilseed crop that is suitable for jet fuel production due to its fatty acid (FA) composition (Vaughn *et al.*, 2004; Moser *et al.*, 2009). The average yield of pennycress seeds is 1500 kg ha^–1^ corresponding to 600–1200 l ha^–1^ of oil, which is higher than soybean and camelina that produce 450 l ha^–1^ and 420–640 l ha^–1^, respectively (Moser *et al.*, 2009). In earlier studies, the biodiesel derived from pennycress oil has shown excellent physical properties that met the requirement of the fuel standard for the American Society for Testing and Materials (Moser *et al.*, 2009; Moser, 2012). Additionally, a recent life cycle assessment has further demonstrated that pennycress-derived fuels could qualify as advanced biofuel with >50% reductions in greenhouse gas emission compared with petroleum (Fan *et al.*, 2013). These findings together highlight the potential of pennycress oil as an alternative source of jet fuel. However, in order to make pennycress an economically viable source of jet fuel, it is important to consider improving the current oil content (35% of the seed biomass) by metabolic engineering and/or breeding.

In embryos, biomass components are synthesized from carbons received from the mother plant. In fact, in oilseeds, the conversion of carbons from substrates into oil can be costly due to a substantial loss of carbon as CO_2_ (Goffman *et al.*, 2005). Indeed, the carbon precursor for *de novo* FA synthesis in the plastid is acetyl-CoA, which is generated from the oxidative decarboxylation of pyruvate through the pyruvate dehydrogenase complex (Johnston *et al.*, 1997). As a result, one of the three carbons entering fatty acid synthesis is lost as CO_2_, making oil production less efficient in terms of the overall carbon economy. Thus, the measurement of carbon utilization provides a way to evaluate metabolic efficiency of a particular tissue, and it is calculated as the ratio of the carbons stored in biomass to those imported from substrates (Chen and Shachar-Hill, 2012). Furthermore, carbon conversion efficiency (CCE) indicates not only differences in metabolism of plant organs but also a potential to improve the biomass components such as oil content.

Several studies measured CCEs of embryos from agriculturally relevant oilseed crops such as maize (Alonso *et al.*, 2010*a*; Cocuron *et al.*, 2019*b*), soybean (Allen *et al.*, 2009), rapeseed (Goffman *et al.*, 2005), and sunflower (Alonso *et al.*, 2007*a*). In these works, carbon balancing was used to estimate the efficiency with which embryos convert substrate carbons into biomass by taking advantage of *in vivo* culture conditions that mimic the development of the embryo *in planta*. However, establishing culture conditions involved characterizing metabolites that embryos received in nature as carbon and nitrogen sources from the mother plants. For immature seeds containing liquid endosperm, compounds that play important roles in embryo metabolism and growth were characterized by the isolation of the endosperm and the analysis of its sugar and amino acid composition (Schwender and Ohlrogge, 2002). Similarly, measuring the CCE for pennycress embryos would require measuring the sugars and amino acids present in the endosperm liquid. In addition to serving as a source of metabolites, the developmental process of the endosperm is highly integrated with the normal physiology of seeds.

In dicots, the embryo is the main reservoir site, and its survival and development depend on the surrounding maternal and endospermic tissues through the assistance of the suspensor. Even though the suspensor develops rapidly and degenerates in the later stages of seed development, it serves as a conduit for the nutrients and growth regulators at an earlier stage by pushing the embryo into the endosperm cavity which contains nutrients [carbon and nitrogen sources, and hormone(s)] acquired from the mother plant (Yeung and Meinke, 1993; Hirner *et al.*, 1998; Kawashima *et al.*, 2009; Melkus *et al.*, 2009). As the embryo matures by feeding on the endosperm, it undergoes several developmental stages reflected by different physical shapes such as early globular, late globular, triangular, heart, late heart, and torpedo stages (Beauzamy *et al.*, 2016). In addition to the nutrients, the endosperm plays an important role in supplying the embryo with hormone(s). Several studies reported that the dissected endosperm is capable of releasing and synthesizing abscisic acid (ABA) to regulate the embryonic growth (Lee *et al*., 2010, 2012).

Considering the aforementioned factors relevant for embryo growth, several studies have successfully established *in vivo* embryo culture conditions for heterotrophic and photoheterotrophic embryos (Schwender and Ohlrogge, 2002; Alonso *et al*., 2007*a*, 2010*a*; Allen *et al.*, 2009). However, none of the previous works took into account the relevance of the hormonal content for culture conditions. The overall goal of this study is to assess the efficiency with which pennycress embryos incorporate carbon into biomass. For this purpose, first the main carbon and nitrogen sources along with hormone(s) from the liquid endosperm were identified and quantified through LC–tandem MS (LC-MS/MS). Then, *in vivo* embryo culture conditions were established and validated by comparing the biomass accumulation and growth rates with those *in planta*. The CCE for pennycress embryos was calculated after determining the carbons from the substrates used for biomass components. Finally, substrates from the media were replaced by ^13^C-labeled substrates in order to unveil the reactions contributing to the CCE in pennycress embryos.

## Materials and methods

### Chemicals

Metabolite standards, 3 N methanolic HCl, toluene, *N*-butylamine, and 1000× Gamborg’s vitamin solution were purchased from MilliporeSigma. [U-^13^C_7_]Benzoic acid, [1,2-^13^C_2_]glucose, [U-^13^C_6_]glucose, [U-^13^C_12_]sucrose, [U-^13^C_5_]glutamine, and [U-^13^C_2_]glycine were obtained from Isotec. Potassium hydroxide (KOH), acetic acid, formic acid, and solvents for GC-MS and LC-MS/MS were purchased from Fisher Scientific. Gibberellins (GA_4_/GA_7_) and Murashige and Skoog (MS) basal salt were ordered from PhytoTechnology Laboratories.

### Plant growth

Pennycress seeds of the Ames 30982 accession were obtained from the North Central Regional Plant Introduction Station. Germination of the seeds, growing of the plant, and tagging of the flowers were conducted as previously described (Tsogtbaatar *et al.*, 2015).

### Endosperm analysis

#### Endosperm collection

The liquid endosperm from seed at 10–11 days after pollination (DAP) was collected under a dissecting microscope using a 3/10 ml insulin syringe. A total of 5–10 µl of endosperm was harvested in a 1.5 ml tube kept on ice. Each tube was centrifuged at 4 °C at 17 000 *g* for 5 min. The volume of each endosperm was measured using a 10 µl Hamilton syringe, and was transferred to a pre-chilled 2 ml screw-cap tube containing 100 nmol of [U-^13^C_12_]sucrose and 10 nmol of [U-^13^C_2_]glycine for quantification of sugars and amino acids, respectively. For the analysis of hormones, the same steps as mentioned above were followed, except that 5 nmol of [U-^13^C_7_]benzoic acid was added as internal standard. Finally, samples were flash-frozen in liquid nitrogen and stored at –80 °C.

#### Sugar and amino acid extraction

A 0.5 ml volume of boiling water was added to each tube, and the biological extracts were immediately incubated at 100 °C for 10 min. Then, the tubes were transferred on ice and centrifuged at 17 000 *g* at 4 °C for 1 min. Supernatants were freeze-dried in a lyophilizer overnight. Dried extracts were re-suspended with 0.5 ml of ultrapure water, then loaded on a 0.22 ìm Nanosep filtering devices, and centrifuged at 17 000 *g* at 4 °C for 5 min. Eluates were stored in a –80 °C freezer until LC-MS/MS analysis.

#### Hormone extraction

Hormones were extracted as previously described (Forcat *et al.*, 2008). Briefly, 100 µl of a solution of methanol:water (10:90, v/v) containing 1% acetic acid was added to 5 µl of each pennycress endosperm containing 5 nmol of [U-^13^C_7_]benzoic acid.

#### Sugar, amino acid, and hormone quantification

Sugars and amino acids from pennycress endosperm were diluted by 100, then separated and quantified through LC-MS/MS as previously described (Cocuron *et al.*, 2014; Tsogtbaatar *et al.*, 2015). Hormone extracts were analyzed as previously published (Cocuron *et al.*, 2019*a*) with minor modifications. Transitions for ABA, gibberellic acid (GA_3_), and [U-^13^C_7_]benzoic acid were added to the pre-existing multiple reaction monitoring (MRM) method (Table 1). The quantities of sugars, amino acids, and hormones were determined using the aforementioned internal standards and a known concentration of external standards.

**Table 1.** Mass spectrometry parameters for the detection of hormones

Metabolite	Precursor ion	Product ion	RT (min)	DP (V)	EP (V)	CE (V)	CXP (V)
Abscisic acid	263	153	6.8	-95	-10	-18	-17
Gibberellic acid (GA_3_)	345	143	3.8	-155	-10	-54	-18
Salicylic acid	136	92	8.5	-80	-10	-26	-39
[U-^13^C_7_]Benzoic acid	128	83	6.6	-40	-10	-16	-33

RT, retention time; DP, declustering potential; EP, entrance potential; CE, collision energy; CXP, collision cell exit potential, are shown.

### Medium composition and embryo culture conditions

Siliques from pennycress at 10 DAP were collected, sterilized using a 20% bleach solution for 5 min, and rinsed five times with sterile water under aseptic conditions. Siliques were dissected under a microscope and a total of eight embryos were transferred to a six-well tissue culture plate containing a double-glass fiber filter soaked with 1 ml of medium consisting of 80 mM glucose, 35 mM glutamine, 10 mM HEPES (pH 6.3), 4.3 g l^–1^ MS basal medium, 10% polyethylene glycol (PEG; 4000), 1000× Gamborg’s vitamin solution, and 6 µM ABA. Embryos were incubated under a light intensity of 20 µmol m^–2^ s^–1^ for 6 d at 21 °C. The culture plates were covered with a green film. Finally, embryos were harvested at 16 DAP, rinsed with ultrapure water, flash-frozen in liquid nitrogen, lyophilized for 3 d, and stored at –80 °C until further analysis.

### Biomass extraction

Oil, proteins, and starch were sequentially extracted as previously described (Cocuron *et al.*, 2014). A 1:5 dilution was applied to fatty acid methyl ester (FAME) samples. The remaining pellet after oil and protein extraction was considered to represent the carbohydrate content.

### Protein hydrolysis and amino acid purification

Proteins were hydrolyzed following a protocol previously published (McClure *et al.*, 2017).

### Quantification of biomass components

FAME, protein, and starch extracts were quantified according to previously published methods (Cocuron *et al.*, 2014; Tsogtbaatar *et al.*, 2015).

### Quantification of proteinogenic amino acids through LC-MS/MS

Proteinogenic amino acid samples were dissolved in 500 µl of 0.01 N HCl and analyzed through LC-MS/MS as previously described (McClure *et al.*, 2017).

### Determination of the carbon conversion efficiency

CCE was calculated as follows:

$$\begin{array}{l} CCE\;\left(\%\right)=\frac{total\;carbon\;into\;biomass\;\left(\mu mol\;per\;embryo\right)}{total\;carbon\;uptake\;\left(\mu mol\;per\;embryo\right)}\times 100 \end{array}$$ (1)

### Carbon uptake

Carbon uptake was estimated as the difference between initial quantities of substrates and the remaining quantities in the medium after embryo incubation. For this purpose, embryos at 10 DAP were cultured as previously mentioned, and, in parallel, culture plates were set up with the medium alone (no embryos). After 6 d of incubation, the embryos were collected, and 1 ml of a standard mixture containing 20 mM [U-^13^C_6_]glucose and 30 mM [U-^13^C_2_]glycine was added into each well, and into the wells with medium alone (no embryos). The resulting mixture of the medium and standards from each well was transferred into a 15 ml Falcon tube. The filter papers were rinsed twice with 2 ml of ultrapure water, and combined with the standard and medium into the 15 ml tubes. A 25 µl aliquot of sample was transferred into a 1.5 ml microcentrifuge tube containing 475 µl of ultrapure water. The extract was filtered through a 0.22 µm Nanosep device by spinning at 17 000 *g* for 15 min at 4 °C. The resulting eluate was quantified by LC-MS/MS as previously described (Cocuron *et al.*, 2014; Tsogtbaatar *et al.*, 2015). A 1:100 and 1:250 dilution was applied to each sample prior to analysis of glucose and glutamine consumption, respectively. The quantities of glucose and glutamine remaining in the culture medium after 6 d of incubation were calculated by using internal standards of [U-^13^C_6_]glucose and [U-^13^C_2_]glycine for normalization purposes, and known quantities of the corresponding external standards. The consumption of glucose and glutamine was determined by the difference between their respective initial and remaining concentrations. Finally, the values for substrate uptake were used to calculate the total carbon uptake (µmol per embryo) by Equation 2, and then expressed in nmol embryo^–1^ h^–1^.

$$\begin{array}{ll} Total\;C\;uptake\;(\mu mol\;per\;embryo)\\ & \;=Gln\;consumed\;(\mu mol\;per\;embryo)\times 5\\ & \;+GLC\;consumed\;(\mu mol\;per\;embryo)\times 6 \end{array}$$ (2)

### Carbon into biomass

For each biomass component, the following were calculated: (i) the accumulated amount (g) over a span of 6 d of culture (from 10 to 16 DAP); (ii) the average molecular weight; (iii) the number of moles per embryo (µmol per embryo); and (iv) total carbon number. The total carbon converted into biomass was then determined by Equation 3.

$$\begin{array}{ll} Total\;C\;into\;biomass\left(oil,\;protein,\;and\;carbohydrates\right)\\ & \;=\sum [each\;biomass\;component\left(\mu mol\;per\;embryo\right)\\ & \;\times \;total\;C\;in\;the\;component] \end{array}$$ (3)

Note that the C into each biomass component was calculated using Equations 4–6. The total carbon number (µmol C per embryo) converted into oil was calculated as follows:

$$\begin{array}{l} \begin{array}{ll} & \mu \;moles\;C\;per\;embryo\\ & =\overset{}{\sum }\left(\frac{abundance\;of\;each\;FA\;\left(g\right)}{MW\;of\;each\;FA\left(\frac{g}{mol}\right)}\right)\times \frac{10^{6}}{n}\;\\ & \;\times \overset{}{\sum }(fraction\;of\;each\;FA\;in\;oil\\ & \;\times \;carbon\;number\;of\;the\;respective\;FA\;molecule) \end{array} \end{array}$$ (4)

where *n*=number of embryos; MW=molecular weight.

To calculate the total C into proteins, each amino acid (AA) from storage proteins was quantified in grams (g) using the amino acid composition determined from hydrolyzed proteins (see Supplementary Table S1 at *JXB* online). It is important to note that the molecular weight of each amino acid was calculated considering a water loss (18 g mol^–1^). Finally, the total carbon number (µmol C per embryo) converted into proteins was calculated as follows:

$$\begin{array}{l} \begin{array}{ll} & \mu \;moles\;C\;per\;embryo\\ & \;=\overset{}{\sum }\left(\frac{abundance\;of\;each\;AA\;\left(g\right)}{MW\;of\;each\;AA\;(g\;mol^{\text{\textasciitilde{}}\text{-}1})}\right)\times \frac{10^{6}}{n}\;\\ & \;\times \overset{}{\sum }(fraction\;of\;each\;AA\;in\;protein\\ & \;\times \;carbon\;number\;of\;each\;AA\;molecule) \end{array} \end{array}$$ (5)

To determine the total C into carbohydrates, the molecular weights and the total carbon numbers for starch and cell wall were considered to be those of the glucose monomer. Thus, the values for molecular weight and the total carbon number were 162 g mol^–1^ (considering water loss during polymerization) and 6, respectively.

$$\begin{array}{ll} \mu \;moles\;C\;per\;embryo\\ & \;=\overset{}{\sum }\left(\frac{abundance\;of\;each\;carbohydrate\;\left(g\right)}{\;162\;g\;mol^{\text{-}1}}\right)\\ & \;\times \frac{10^{6}}{n}\times 6 \end{array}$$ (6)

### Embryo cultures with ^13^C-labeled isotopes

^13^C-labeling experiments of pennycress embryos were performed by replacing the sugar and amino acid in the medium described above with the following ^13^C-labeled substrates: (i) 20% [U-^13^C_6_]glucose and 80% [1,2-^13^C_2_]glucose; (ii) 100% [U-^13^C_5_]glutamine; and (iii) 20% [U-^13^C_5_]glutamine and 20% [U-^13^C_6_]glucose. Each labeling experiment was conducted with three biological replicates.

### Extraction of ^13^C-labeled intracellular metabolites and biomass components

#### ^13^C-Labeled intracellular metabolites

Free ^13^C-labeled intracellular metabolites were extracted from each tube containing eight dried 16 DAP embryos using boiling water as previously reported (Koubaa *et al.*, 2013; Cocuron and Alonso, 2014; Cocuron *et al*., 2014, 2017). Note that no ^13^C-labeled internal standards were added at the time of extraction. The extracts were freeze-dried, and stored at –20 °C until further analysis.

#### [^13^C]Lipids and [^13^C]starch

[^13^C]Lipids and [^13^C]starch were sequentially extracted from eight dried embryos and stored at –20 °C as previously described (Tsogtbaatar *et al.*, 2015).

### Analysis of ^13^C labeling in intracellular compounds and biomass

#### Quantification of ^13^C-labeled metabolites

[^13^C]Water-soluble metabolites were resuspended in 300 µl of ultrapure water. The mass isotopomer distribution (MID) of [^13^C]glucose was analyzed by LC-MS/MS using the same gradient and MRM transitions as those previously reported (Cocuron *et al.*, 2014). Additionally, [^13^C]sucrose was enzymatically cleaved into [^13^C]fructose and [^13^C]glucose to assess the labeling of cytosolic hexose phosphates including glucose 6-phosphate and fructose 6-phosphate using LC-MS/MS as described elsewhere (Cocuron *et al.*, 2020).

The labeling of the free [^13^C]amino acid fraction was analyzed by LC-MS/MS utilizing scheduled MRM and the same transitions as those previously published (Cocuron *et al.*, 2017). The labeling abundances of ^13^C-labeled phosphorylated compounds and ^13^C-labeled organic acids were assessed by LC-MS/MS following methods previously published (Alonso *et al.*, 2010*b*; Koubaa *et al.*, 2013; Cocuron and Alonso, 2014).

#### Quantification of [^13^C]lipids by GC-MS

[^13^C]Lipids were extracted and derivatized using *N*-butylamine (Allen *et al.*, 2007). Dried fatty acid butyl amide (FABA) derivatives were resuspended in 500 µl of hexanes and transferred into a GC-MS vial containing an insert. A 1 µl aliquot of each sample was injected and analyzed using a Thermo Trace 1310 gas chromatograph coupled to a single-quadrupole ISQ mass spectrometer. FABAs were separated using a TG-5MS capillary (30 m×0.25 mm×0.50 µm) column from Thermo Fisher Scientific at a constant flow rate of 1.4 ml min^–1^. Helium was used as the carrier gas. The GC conditions were as follows: initial temperature was set to 200 °C, and held for 30 s. The oven temperature was raised to 265 °C at 100 °C min^–1^, and held for 8 min. A second ramp was applied at a rate of 100 °C min^–1^ to reach a final temperature of 300 °C, which was held for 6 min. The injection temperature was fixed at 300 °C and the injection was set to split mode with a split ratio of 50. For the MS analysis, the mass spectra were acquired using electron impact ionization in positive ion mode. Ion source and the interface temperatures were set to 280 °C and 325 °C, respectively. Total ion chromatograms were obtained for the mass range of 40–450 amu with a scan time of 0.071 s to determine retention times of FABA derivatives. Subsequently, selected ion monitoring was utilized to specifically follow: (i) the molecular ions of 311 amu and 393 amu which correspond to butyl amide derivatives of palmitic and erucic acids, respectively; and (ii) 115, 116, and 117 amu representing the M+0, M+1, and M+2 mass isotopomers of the McLafferty fragments resulted from butyl amide derivatization of palmitic and erucic acids. GC-MS data were acquired and processed using Xcalibur software.

#### Quantification of ^13^C labeling in starch by LC-MS/MS

MIDs in ^13^C-labeled starch glucosyl units were analyzed through LC-MS/MS (refer to ‘Quantification of ^13^C-labeled metabolites’). Briefly, 250 µl of each extract containing ^13^C-labeled glucosyl monomers in 0.1 M acetate buffer was transferred onto a 3 kDa Amicon filtering device and centrifuged at 14 000 *g* for 30 min at room temperature. A 10 µl aliquot of the filtered aliquot was diluted with 990 µl of acetonitrile/water (60:40, v/v) in a LC-MS/MS vial. The LC and MS conditions were the same as those of free hexoses in the section ‘Quantification of ^13^C-labeled metabolites’ and previous work (Cocuron *et al.*, 2014).

### Correction for natural abundances

In order to quantify the abundance of each mass isotopomer of metabolites, a correction was performed using Scilab, an open source software (www.scilab.org), for masses and natural abundances of the isotopes of C, N, H, O, and S atoms that are not part of the carbon backbone of the metabolites. Metabolites from unlabeled embryo cultures were corrected using Scilab to assess accuracy of their measured natural abundance of labeling.

### Statistical analysis

Two-tailed, type 3 Student’s tests (*t*-test) were performed, considering statistically significant those *P*-values below 0.05.

## Results and discussion

### Identification of the substrates received by developing pennycress embryos

Establishing culture conditions that resemble the development of *in planta* pennycress embryos is pivotal to reveal the biosynthetic efficiency. Achieving the right conditions involves thorough balancing of the substrate composition, osmotic pressure, and light intensity, all of which collectively influence the embryo development and biomass compositions. Culture conditions are validated when the dry weight and the rates of biomass accumulation of cultured embryos are in agreement with those of *in planta* embryos. To establish the culture conditions that successfully resemble the *in planta* environment of the pennycress embryo, it was essential to characterize the composition of the liquid endosperm that naturally sustains the development of the embryo. Hence, the liquid endosperm was collected at 10 DAP, and sugar, sugar alcohol, amino acid, and hormone contents were quantified by LC-MS/MS (Supplementary Tables S2–S4).

The main sugars in the endosperm were found to be glucose, fructose, and sucrose, with average concentrations of 49.34±5.06, 34.92±4.86, and 6.59±2.29 mM, respectively. Other sugars and sugar alcohols had concentrations below 1 mM (Supplementary Table S2). Even though both fructose and glucose are the major sugars determined in the endosperm, they both serve as substrates for the glycolytic pathway of central carbon metabolism. Because glucose is located slightly upstream, serving as a precursor for several additional pathways such as cell wall and starch, it was chosen as the sole carbon source in the culture medium by replacing fructose. Additionally, the potential action of invertases was determined by measuring [U-^13^C_6_]glucose and [U-^13^C_6_]fructose that might result from the cleavage of [U-^13^C_12_]sucrose added to the endosperm samples. Only traces of [^13^C]hexoses were detected through LC-MS/MS, demonstrating that invertases did not contribute to the pool of fructose and glucose quantified in the liquid endosperm (data not shown).

The analysis of amino acids revealed that the most abundant was glutamine with an average concentration of 40.42±12.82 mM, representing 54.7% of the total amino acids (Supplementary Table S3). The other major amino acids included alanine, threonine, proline, and valine with average concentrations of 3.45±0.17, 4.20±0.62, 3.28±0.18, and 3.48±0.18 mM, respectively. However, the concentrations of these amino acids were ~10 times lower than that of glutamine. Hereafter, glutamine was identified and supplemented as the main amino acid, serving as a nitrogen source for *in vivo* embryo cultures.

Because ABA has been known to influence both embryo development and endosperm cellularization (Lee *et al*., 2010, 2012; Cheng *et al.*, 2014, ABA along with other hormones such as salicylic (SA) and GA were quantified by LC-MS/MS. ABA and SA were the main hormones present in the liquid endosperm, with average concentrations of 0.369±0.005 µM and 0.278±0.048 µM, respectively. SA is known to be involved in plant defense in addition to plant growth, and hence it was not included in the culture media for the embryos (War *et al.*, 2011). ABA was selected as the main hormone, preventing precocious root development while allowing the normal growth of the pennycress embryo (Rai *et al.*, 2011).

### Establishing and validating *in vivo* embryo culture conditions

**Fig. 1.**
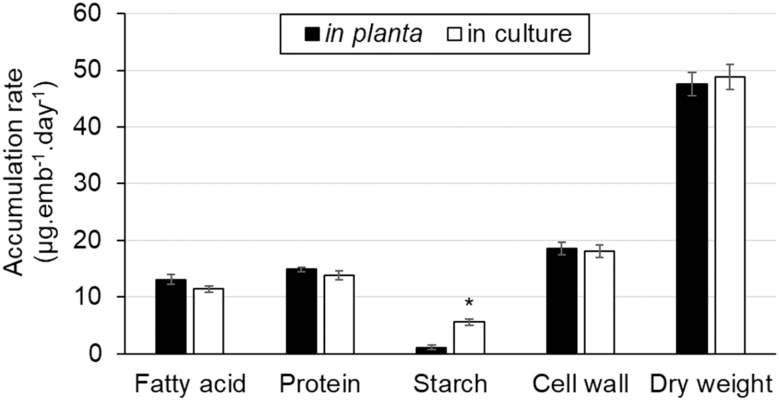
Average rates of biomass accumulation in pennycress embryos. The average rates of biomass synthesis were determined from *in planta* and cultured embryos, and expressed in µg per embryo d^–1^ (*n*=3). A statistically significant (*P*<0.05) value is indicated by an asterisk.

From endosperm analysis, the main substrates and hormone for the developing embryos were determined to be glucose, glutamine, and ABA, respectively (Table 2). In order to establish the *in vivo* embryo culture conditions that mimic *in planta* metabolism, a variety of culture conditions with different concentrations of substrates, light level, and PEG were tested. The ranges assayed were 60–120 mM for glucose, 30–60 mM for glutamine, 0–20 µM for ABA, and 0–20% for PEG concentrations, and 10–30 µmol m^–2^ s^–1^ for light intensity. After 6 d of incubation, the dry weights and the rates of biomass accumulation of cultured embryos were compared with those *in planta*. The condition that resulted in embryos with physiological growth and biomass synthesis rates similar to *in planta* embryos was a medium containing 80 mM glucose, 35 mM glutamine, 6 µM ABA, 10% PEG, and a light intensity of 20 µmol m^–2^ s^–1^ (Fig. 1). Through the comparisons between various oilseed embryo cultures (Schwender *et al*., 2003, 2006; Goffman *et al.*, 2005; Allen *et al*., 2007, 2009; Alonso *et al*., 2007*a*, 2010*a*; Pollard *et al.*, 2015; Cocuron *et al.*, 2019*b*), it was shown that glucose and glutamine are the most common constituents in media besides other additional components such as fructose, sucrose, alanine, and asparagine. These results are in agreement with our finding. Nevertheless, pennycress embryos grown in culture without ABA had a biomass rate 50% higher than *in planta* (data not shown). ABA is known to regulate seed size (Cheng *et al.*, 2014), and for this specific reason it was considered and included in our study.

**Table 2.** Main substrates and hormone from pennycress endosperm

Metabolite	Concentration (mM)
Glucose	49.34±5.06
Fructose	34.92±4.86
Glutamine	40.42±12.82
ABA	0.37×10^–3^±0.01×10^–3^

The metabolites were quantified using an MRM scan survey as indicated in the Materials and methods. The concentrations of metabolites are the average ±SD of three biological replicates (*n*=3).

In these culture conditions, carbohydrates were accumulating significantly faster in pennycress embryos in culture (23.65±1.62 µg per embryo d^–1^) than *in planta* (19.61±0.85 µg per embryo d^–1^; Fig.1). In fact, this phenomenon of higher starch synthesis has previously been observed in cultured sunflower embryos which are heterotrophic (Alonso *et al.*, 2007*a*). Additionally, the FAs obtained from the *in vivo* and *in planta* embryos were very similar (Supplementary Fig. S1), though palmitic, linoleic, and eicosadienoic acids were significantly higher, and erucic acid was significantly lower in the cultured embryos. Such a discrepancy in the FA profile has previously been observed in Arabidopsis cultured embryos, where an increase in the accumulation of linolenic acid was accompanied by a decrease in long chain FAs (Lonien and Schwender, 2009). Nevertheless, the rates and total content of FAs were not significantly different, suggesting that the carbon flow towards major carbon sinks was not affected.

### Pennycress embryos convert carbon into biomass with high efficiency

**Fig. 2.**
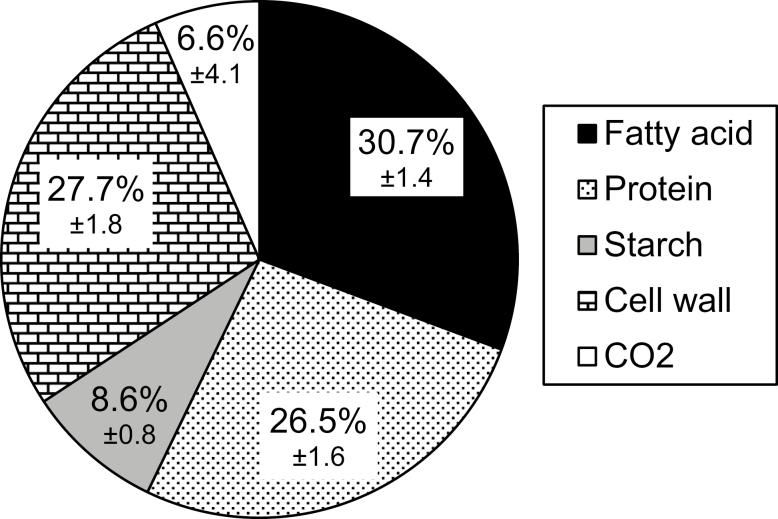
The percentage distribution of carbon among biomass fractions. The highest to the lowest carbon sinks (mol C per embryo) are in the order of fatty acid (black), cell wall (bricks), protein (dots), and starch (gray). The difference between carbons in the consumed substrates and in biomass components represents the carbons released as CO_2_ (white). The overall CCE is 93.4%. Values are the average of three biological replicates ±SD.

Carbon mass balancing is a common method to determine the efficiency with which microorganisms (Loubiere and Novak, 2000; Converti and Perego, 2002; Saez *et al*., 2002) and plants (Schwender *et al.*, 2004; Goffman *et al.*, 2005; Alonso *et al*., 2007*a*, 2010*a*, 2011; Allen *et al.*, 2009) convert substrate metabolites into final products. This approach involves measuring the total carbon content in substrates, biomass, and CO_2_. Glucose and glutamine consumption by the cultured pennycress embryos was first quantified through LC-MS/MS. The total carbon uptake was determined to be 14.48 µmol C per embryo, of which 12.21 µmol C was from glucose and 2.27 µmol C was from glutamine. The abundances of biomass components were measured, and then their quantities were expressed in C atoms converted into biomass using the equations described in the Materials and methods. The total C incorporated into biomass was 13.52 µmol C embryo, of which 4.44, 3.83, and 5.25 were converted into FAs, proteins, and carbohydrates, respectively. Using the values for the total C uptake and C converted into biomass, it was shown that pennycress embryos convert substrates into biomass components with an efficiency of 93.4% (Fig. 2). This CCE of 93.4% is substantially higher in pennycress than in other photoheterotrophic oilseed embryos such as rapeseed (86%), soybean (83%), and camelina (57%) (Goffman *et al.*, 2005; Schwender *et al.*, 2006; Allen *et al.*, 2009; Chen and Shachar-Hill, 2012). This means that to improve oil content in pennycress, carbon flow would have to be redirected from other biomass components, such as proteins and/or carbohydrates.

The carbon precursor for *de novo* FA synthesis in the plastid is acetyl-CoA, which is generated from the oxidative decarboxylation of pyruvate through the pyruvate dehydrogenase complex (Johnston *et al.*, 1997). As a result, one of the three carbons entering FA synthesis is lost as CO_2_, and the ratio of carbon to oil over CO_2_ is expected to be 2:1 (Schwender *et al.*, 2004; Goffman *et al.*, 2005). It was shown that a ratio higher than 2 was the indication of the occurrence of other non-conventional pathways involved in oil production (Schwender *et al*., 2004, 2006). In the case of pennycress embryos, this ratio was found to be 4.7, implying that non-conventional pathways are likely to be responsible for such a high biosynthetic efficiency (Supplementary Table S5). To unveil the underlying reactions by which pennycress embryos achieve such a high CCE, parallel labeling experiments were performed on embryos cultured with ^13^C-labeled glucose or ^13^C-labeled glutamine.

### Confirmation of isotopic steady state

Isotopic steady state was confirmed by replacing unlabeled glucose and glutamine present in the culture medium with 20% [U-^13^C_6_]glucose and 20% [U-^13^C_5_]glutamine. When isotopic steady state is reached, the majority of intracellular metabolites have an average carbon labeling of 20% (Cocuron *et al.*, 2019*b*). Metabolites were extracted and their labeling was determined as described in the Materials and methods. For each compound, the average carbon labeling was calculated as a percentage, using the following equation:

$$\begin{array}{ll} Average\;carbon\;labeling\left(\%\right)\\ & \;=\frac{\left(M+1\right)\times 1+\ldots +(M+n)\times n}{n} \end{array}$$ (7)

where (M+*n*) represents the percentage abundances of M+*n* mass isotopomers, and *n* is the number of carbon atoms that can be labeled in a given molecule. The resulting labeling data showed that of 31 intracellular metabolites, 25 reached isotopic steady state with an average ^13^C labeling of 20%, four had labeling dilutions (average ^13^C labeling <18%) most probably due to the presence of a metabolically inactive pool, and two had impurities in the LC-MS/MS run (average ^13^C labeling >23%; Supplementary Table S6). Therefore, the compounds with average labeling abundances >23% per carbon (alanine and threonine) were omitted from further consideration. However, those with lower than 18% enrichment per carbon (glutamate, serine, succinate, and the cytosolic acetyl-CoA unit) were retained after applying a correction factor as previously described (Cocuron *et al.*, 2019*b*).

Upon confirming isotopic steady state, embryos were cultured in a mixture of [1,2-^13^C_2_]glucose and [U- ^13^C_6_]glucose in order to provide information on the carbon rearrangements of metabolites in glycolysis and the oxidative pentose phosphate pathway (OPPP; Libourel *et al.*, 2007). Coverage of organic acids and tricarboxylic acid (TCA) cycle-derived amino acids was assessed by replacing unlabeled glutamine in the culture medium by [U-^13^C_5_]glutamine. The labeling data from these two parallel labeling experiments give complementary information which allows a good coverage of the metabolic network (Schwender *et al.*, 2006; Alonso *et al*., 2007*a*, 2010*a*, 2011; Allen *et al.*, 2009; Cocuron *et al.*, 2019*b*).

### Labeling with [^13^C]glucose

**Fig. 3.**
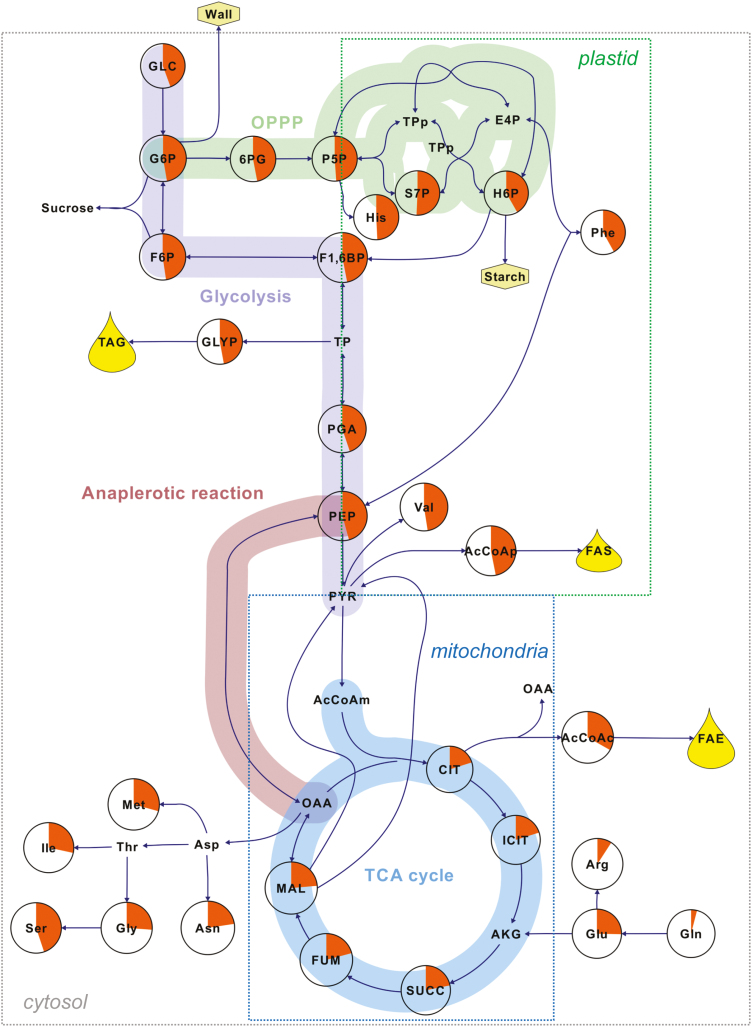
The average labeling per carbon (%) in intracellular compounds after incubating pennycress embryos with [^13^C]glucose. ^13^C atoms incorporated in sugars, amino acids, phosphorylated compounds, and organic acids were determined and measured by LC-MS/MS. The average labeling per carbon for each metabolite was calculated as a percentage from three biological replicates. The percentages are represented by orange pie charts. The boxes labeled plastid, mitochondria, and cytosol include metabolites that are specifically synthesized in these compartments. Abbreviations (in alphabetical order): AcCoA(p, c, m), (plastidic, cytosolic, mitochondrial) acetyl-CoA; AKG, α-ketoglutarate; CIT, citrate; E4P, erythrose 4-phosphate; F1,6BP, fructose 1,6-bisphosphate; F6P, fructose 6-phosphate; FAE, fatty acid elongation; FAS, fatty acid synthesis; FUM, fumarate; G6P, glucose 6-phosphate; GLC, glucose; GLYP, glycerol phosphate; H6P, hexose 6-phosphates; ICIT, isocitrate; MAL, malate; OAA, oxaloacetate; PEP, phosphoenolpyruvate; 6PG, 6-phosphogluconate; PGA, phosphoglycerate; P5P, pentose 5-phosphates; PYR, pyruvate; S7P, sedoheptulose 7-phosphate; SUCC, succinate; TAG, triacylglycerols; and TP(p), (plastidic) triose phosphates.

MIDs were determined from the analysis of labeling in intracellular metabolites through LC-MS/MS (Tables 3–6). As expected, intermediates from glycolysis and the OPPP showed higher values corresponding to the average labeling abundance per carbon than those from the TCA cycle and the amino acids derived from it (Fig. 3). All mass isotopomers (M+0 to M+6) were observed and measured for fructose 1,6-bisphosphate (F1,6BP) (Table 3). Even though M+2 and M+6 were expected due to the isotopic composition of the substrates ([1,2-^13^C_2_]- and [U-^13^C_6_]glucose, the presence of other mass isotopomers in F1,6BP, such as M+0, M+3, M+4, and M+5, indicates the reversibility of fructose bisphosphate aldolase (EC 4.1.2.13). This enzyme resynthesizes F1,6BP from two triose phosphates (glyceraldehyde 3-phosphate and dihydroxyacetone phosphate). On the other hand, the presence of the M+1 isotopomer can be explained by the occurrence of the OPPP. When glucose 6-phosphate (G6P) with labeling on C1 and C2 (as in the case of [1,2-^13^C_2_]glucose) undergoes oxidative reactions, it loses its C1 as CO_2_ and produces pentose phosphates with labeling on C1. The resulting pentose phosphate then undergoes non-oxidative reactions of the PPP, forming F1,6BP with a labeling on one carbon (Table 3). The subcellular localization of the OPPP, a key pathway producing NADPH, was investigated by comparing the MID of 6-phosphogluconate (6PG), the product of the first oxidative reaction of the OPPP, with those of hexose phosphates from the cytosol and plastid (Table 4). For this purpose, sucrose and starch were enzymatically cleaved into their hexose monomers to reveal the labeling pattern in the cytosol and plastid, respectively. According to the MID values listed in Tables 3 and 4, 6PG is closer to that of cytosolic G6P, indicating that the oxidative part of the PPP is more active in the cytosol than in the plastid. Therefore, in developing pennycress embryos, the OPPP produces NADPH in the cytosol, which may be used as reductant power for fatty acid elongation.

**Table 3.** MID and average labeling abundance (%) per carbon of phosphorylated compounds.

Phosphorylated compounds	Mass isotopomers	Average labeling (% ^13^C abundance)	
		[^13^C]GLC	[^13^C]Gln
PGA	M+0	40.12±0.88	96.92±0.33
	M+1	8.99±0.41	3.42±0.38
	M+2	29.57±0.43	0.00±0.25
	M+3	22.13±0.76	0.21±0.28
6PG	M+0	6.13±0.64	92.12±0.76
	M+1	9.10±0.57	8.76±0.68
	M+2	40.72±1.22	0.00±0.25
	M+3	14.82±0.98	0.02±0.28
	M+4	10.33±0.63	0.00±0.25
	M+5	10.26±0.75	0.00±0.25
	M+6	9.08±0.48	0.00±0.25
F1,6BP	M+0	8.45±0.67	93.27±0.50
	M+1	7.71±0.48	6.63±0.43
	M+2	37.91±0.33	0.00±0.25
	M+3	15.70±0.77	0.11±0.31
	M+4	11.40±0.58	0.08±0.30
	M+5	10.56±0.87	0.07±0.36
	M+6	8.81±0.76	0.11±0.33
GLYP	M+0	36.61±2.42	96.40±0.51
	M+1	8.98±0.57	3.96±0.41
	M+2	33.11±2.53	0.00±0.00
	M+3	22.05±0.94	0.08±0.28
P5P	M+0	11.70±0.66	94.36±0.47
	M+1	16.89±1.11	5.75±0.45
	M+2	27.90±0.70	0.00±0.00
	M+3	15.06±0.47	0.13±0.31
	M+4	18.23±0.84	0.01±0.27
	M+5	10.75±0.55	0.00±0.26
PEP	M+0	38.36±0.75	96.54±0.72
	M+1	9.16±0.67	3.30±0.53
	M+2	31.41±0.67	0.00±0.39
	M+3	21.79±0.94	0.53±0.39
S7P	M+0	4.04±0.38	92.62±0.63
	M+1	7.28±0.57	7.48±0.57
	M+2	19.29±0.52	0.00±0.00
	M+3	20.28±0.91	0.06±0.28
	M+4	19.73±0.99	0.01±0.25
	M+5	13.78±0.63	0.00±0.25
	M+6	11.20±0.45	0.00±0.25
	M+7	4.76±0.38	0.01±0.26

Each mass isotopomer was detected and measured through LC-MS/MS in negative ion mode. MID and the average labeling abundance (%) in each carbon were determined for phosphorylated compounds from [^13^C]GLC and [^13^C]Gln experiments. The values listed are the average of three biological replicates ±SD.

**Table 4.** MID and average labeling abundance (%) per carbon of compounds specific to the cytosol or plastid

Compartmentalized metabolites	Mass isotopomers	Average labeling (% ^13^C abundance)	
		[^13^C]GLC	[^13^C]Gln
AcCoAp	M+0	49.74±1.04	89.19±0.68
	M+1	10.64±0.48	4.59±0.36
	M+2	41.57±0.83	6.54±0.59
AcCoAc	M+0	59.30±1.14	48.07±0.85
	M+1	15.08±0.63	9.97±0.32
	M+2	25.62±1.84	41.96±1.28
H6Pp	M+0	9.99±0.87	92.16±0.71
	M+1	7.55±1.07	7.31±0.64
	M+2	49.44±6.16	0.52±0.33
	M+3	12.71±1.43	0.02±0.27
	M+4	6.71±2.92	0.00±0.25
	M+5	3.84±3.89	0.01±0.25
	M+6	9.97±0.61	0.00±0.25
G6Pc	M+0	7.96±1.16	92.37±1.14
	M+1	5.53±0.49	6.56±0.46
	M+2	43.30±0.61	0.81±0.97
	M+3	13.70±0.56	0.19±0.32
	M+4	9.75±0.56	0.05±0.27
	M+5	9.21±0.46	0.02±0.27
	M+6	10.92±0.35	0.01±0.26
F6Pc	M+0	7.56±1.21	92.66±0.46
	M+1	5.24±0.46	6.68±0.45
	M+2	42.79±0.59	0.44±0.30
	M+3	14.42±0.66	0.18±0.29
	M+4	9.92±0.55	0.03±0.27
	M+5	9.91±0.51	0.00±0.25
	M+6	10.44±0.47	0.00±0.26
GLCc	M+0	10.14±1.19	92.43±0.46
	M+1	3.08±1.01	6.86±0.43
	M+2	54.69±3.07	0.57±0.31
	M+3	8.42±1.12	0.11±0.33
	M+4	5.08±0.84	0.02±0.27
	M+5	4.57±0.93	0.01±0.26
	M+6	14.23±1.38	0.01±0.26

For the labeling information of plastidic and cytosolic acetyl-CoA, McLafferty fragments of palmitic (C16:0) and erucic acids (C22:1) were analyzed, respectively, by GC-MS (*n*=3). Starch and sucrose glucosyls were subject to LC-MS/MS to elucidate the labeling of plastidic and cytosolic hexoses, respectively. MID and the average labeling abundance (%) in each carbon were determined for the compounds listed from [^13^C]GLC and [^13^C]Gln experiments. The values are the average of three biological replicates ±SD. Cytosolic and plastidic compounds are denoted as ‘c’ and ‘p’, respectively.

MID of free intracellular glucose (Table 4) revealed all seven mass isotopomers (M+0 through M+6), whereas external glucose only had two mass isotopomers: M+2 and M+6. This observation indicates that the extracellular glucose was not the only source for the intracellular hexose pool. The contribution of cytosolic G6P to intracellular glucose was calculated using Equation 8.

$$\begin{array}{ll} Vg\times GLCext\left(M+3\right)+Vres\times G6P\left(M+3\right)\\ & \;=GLCint\left(M+3\right) \end{array}$$ (8)

where *Vg* and *Vres* correspond to rates of extracellular glucose (GLCext) uptake and resynthesis of intracellular glucose (GLCint) from G6P, respectively. GLCext (M+3), G6P (M+3), and GLCint (M+3) represent the percentage abundances of M+3 mass isotopomers of extracellular glucose, G6P, and intracellular glucose, respectively. In our labeling conditions, GLCext (M+3)=0, and *Vres* was calculated to be 60% using the abundances listed in Tables 3 and 4. This indicates that ~60% of the total intracellular glucose pool was formed from intracellular hexose phosphates in developing pennycress embryos, revealing the occurrence of a substrate cycle.

A substrate cycle, also referred to as futile cycling, is described as a cycle of substrate synthesis and degradation, consuming ATP without apparent functions. The cycle glucose↔G6P can occur via sucrose synthesis and degradation (Dieuaidenoubhani *et al.*, 1995) and/or via a potential glucose 6-phosphatase (Alonso *et al.*, 2005). In fact, this process can be high-energy demanding and the overall ATP consumption varies from 5% to 70%, depending on the plant organ (Hatzfeld and Stitt, 1990; Dieuaidenoubhani *et al.*, 1995; Rontein *et al.*, 2002; Alonso *et al*., 2005, 2007*a*, *b*). According to the measurements on heterotrophic tissues, the glucose re-synthesis in maize embryos (Alonso *et al.*, 2010*a*), maize root tips (Alonso *et al.*, 2005), sunflower embryos (Alonso *et al.*, 2007*a*), and maize endosperm (Alonso *et al.*, 2011) accounted for 45, 86, 38, and 64% of the total glucose pools, respectively. In comparison with heterotrophic embryos accumulating oil, the production of glucose from G6P in pennycress embryos is substantially higher (60%). To date, there are no such measurements available in photoheterotrophic embryos. However, one can anticipate that this high re-synthesis of glucose consumes ATP, but photosynthesis may be able to compensate for it, providing sufficient ATP for accumulating biomass with high CCE.

### Labeling with [^13^C]glutamine

**Fig. 4.**
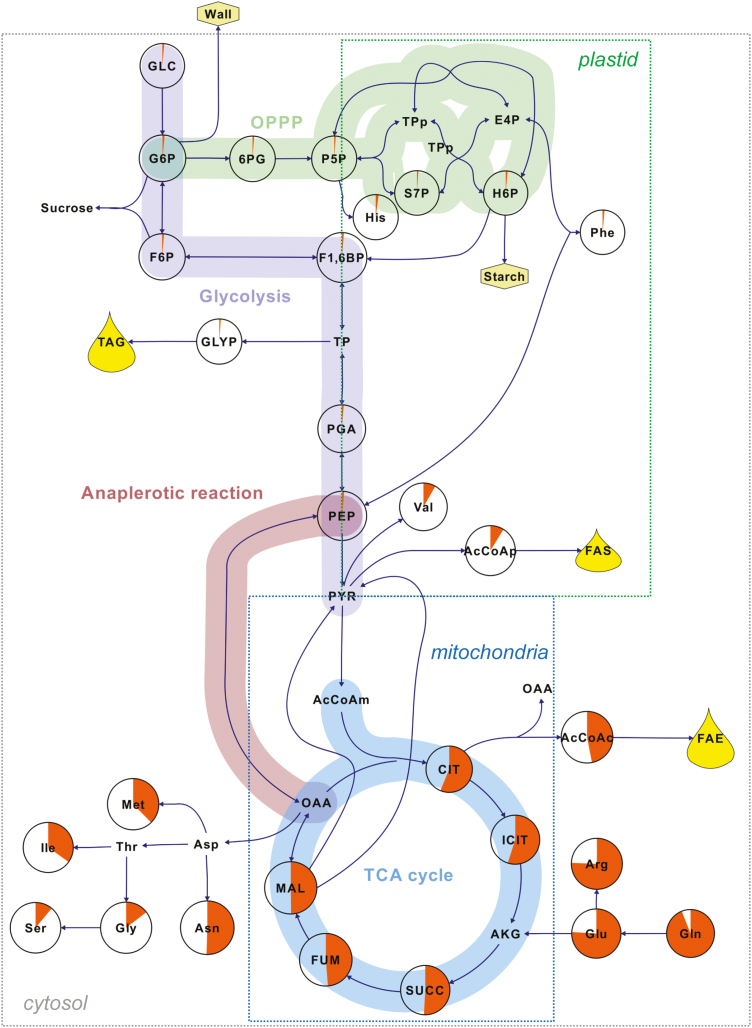
The average labeling per carbon (%) in intracellular compounds after incubating pennycress embryos with [U-^13^C_5_]glutamine. ^13^C atoms incorporated in sugars, amino acids, phosphorylated compounds, and organic acids were determined and measured by LC-MS/MS. The average labeling per carbon for each metabolite was calculated as a percentage from three biological replicates. The percentages are represented by orange pie charts. The boxes labeled plastid, mitochondria, and cytosol include metabolites that are specifically synthesized in these compartment. Abbreviations (in alphabetical order): AcCoA(p, c, m), (plastidic, cytosolic, mitochondrial) acetyl-Co A; AKG, α-ketoglutarate; CIT, citrate; E4P, erythrose 4-phosphate; FAE, fatty acid elongation; FAS, fatty acid synthesis; F1,6BP, fructose 1,6-bisphosphate; F6P, fructose 6-phosphate; FUM, fumarate; GLC, glucose; GLYP, glycerol phosphate; G6P, glucose 6-phosphate; H6P, hexose 6-phosphates; ICIT, isocitrate; MAL, malate; OAA, oxaloacetate; PEP, phosphoenolpyruvate; 6PG, 6-phosphogluconate; PGA, phosphoglycerate; P5P, pentose 5-phosphates; PYR, pyruvate; S7P, sedoheptulose 7-phosphate; SUCC, succinate; TAG, triacylglycerols; and TP(p), (plastidic) triose phosphates.

In contrast to ^13^C-labeled glucose substrates, incubating embryos with [U-^13^C]glutamine resulted in higher abundances of labeling in metabolites from the TCA cycle and its derived amino acid (Fig. 4). Intermediates from glycolysis and the OPPP were labeled at the levels of their natural abundances (1.07%). This result directly indicates no significant activity of gluconeogenesis in developing pennycress embryos. In addition, the average labeling abundance of phosphoenolpyruvate (PEP) is only 1.39±0.33% in comparison with the higher labeling enrichments measured from the TCA cycle intermediates (Fig. 4; Table 6). This demonstrates that phosphoenolpyruvate carboxykinase (EC 4.1.1.32), an enzyme that catalyzes the conversion of oxaloacetate (OAA) into PEP, was not contributing to gluconeogenesis.

**Table 6.** MID and average labeling abundance (%) of organic acids

Organic acids	Mass isotopomers	Average labeling (% ^13^C abundance)	
		[^13^C]GLC	[^13^C]Gln
CIT	M+0	45.38±0.83	18.14±0.99
	M+1	18.90±0.54	6.58±0.48
	M+2	18.56±0.35	6.17±0.52
	M+3	8.37±0.44	11.32±0.52
	M+4	5.37±0.39	16.30±1.03
	M+5	2.73±0.32	33.76±1.20
	M+6	0.76±0.28	8.38±1.00
ICIT	M+0	45.05±1.84	19.37±1.14
	M+1	19.28±0.83	6.97±0.52
	M+2	18.53±1.12	6.13±0.82
	M+3	8.39±0.71	11.60±0.95
	M+4	5.35±0.59	15.78±1.20
	M+5	2.72±0.35	32.04±1.44
	M+6	0.75±0.29	8.74±0.81
MAL	M+0	54.61±1.07	35.82±0.45
	M+1	14.68±0.37	10.12±0.74
	M+2	16.45±0.57	7.87±0.66
	M+3	10.77±0.64	13.71±0.55
	M+4	3.65±0.35	33.04±1.20
SUCC*	M+0	51.27±0.76	36.77±1.73
	M+1	21.36±1.51	6.76±0.69
	M+2	19.93±0.82	12.72±1.40
	M+3	3.60±0.55	4.01±0.93
	M+4	3.85±0.40	39.75±2.25
FUM	M+0	59.20±1.36	37.64±1.13
	M+1	14.05±1.09	9.38±1.19
	M+2	14.29±1.51	7.37±0.77
	M+3	9.42±0.49	13.34±1.04
	M+4	3.14±0.43	32.70±1.21

Each mass isotopomer was detected and measured through LC-MS/MS in a negative ion mode. MID and the average labeling abundance (%) in each carbon were determined for organic acids from [^13^C]GLC and [^13^C]Gln experiments. The values listed are the average of three biological replicates ±SD. An asterisk (*) represents a metabolite whose MID values were corrected according to a dilution factor obtained from the 20% labeling experiment (Supplementary Table S6).

**Fig. 5.**
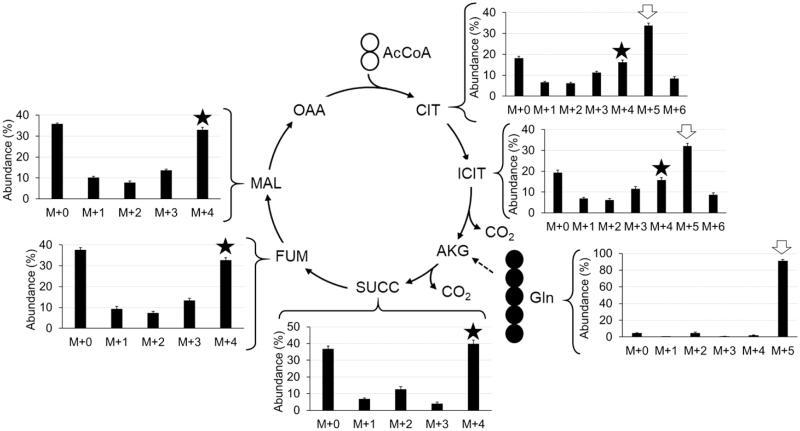
Mass isotopomer distribution in organic acids after labeling pennycress embryos with [U-^13^C_5_]glutamine. ^13^C atoms incorporated into organic acids were detected and measured by LC-MS/MS. Bar graphs representing the abundances (%) of all mass isotopomers are shown next to the corresponding intermediates. Each value is the average, and the error bars are the SD of three biological replicates. The black star and the white arrow highlight M+4 and M+5, respectively. Abbreviations (in alphabetical order): AcCoA, acetyl-Co A; AKG, α-ketoglutarate; CIT, citrate; FUM, fumarate; ICIT, isocitrate; MAL, malate; OAA, oxaloacetate; and SUCC, succinate.

Another important result generated from the [U-^13^C_5_]glutamine experiment is related to the operation of the TCA cycle. According to the conventional activity of the TCA cycle shown in Fig. 5, intracellular glutamine is converted into α-ketoglutarate (AKG; five carbons), then loses one carbon as CO_2_, forming succinyl-CoA (four carbons). As the cycle proceeds from succinate to OAA, the number of carbons in these organic acids does not change (four carbons). However, a new molecule of acetyl-CoA (AcCoA; two carbons) enters the TCA cycle and is condensed with an OAA to produce citrate (CIT; six carbons), which is then converted into isocitrate (ICIT; six carbons). The next step involves production of AKG and CO_2_ from ICIT by isocitrate dehydrogenase (IDH; EC 1.1.1.42). According to the measured MIDs of organic acids (Table 6) and glutamine (Table 5), M+4 was the major labeled mass isotopomer in the four-carbon-containing organic acids, as expected. However, the most abundant mass isotopomer in CIT and ICIT was M+5 (Fig. 5), which indicates that IDH, conventionally regarded as catalyzing a thermodynamically irreversible decarboxylation, is operating reversibly in developing pennycress embryos. Reductive carboxylation of AKG to ICIT through IDH was first observed in rat liver and heart (Des Rosiers *et al.*, 1994; Comte *et al.*, 1997). Since then, the role of this enzyme in FA biosynthesis of tumor and mammalian cells under hypoxia has been reported (Dang *et al.*, 2009; Gaglio *et al.*, 2011; Filipp *et al.*, 2012). However, the discovery of this phenomenon in oilseed embryos and other plant tissues is relatively new; the first report on IDH catalyzing carboxylation of AKG in oilseed was on rapeseed embryos (Schwender *et al.*, 2006). This phenomenon was explained by high demand of the citrate for FA elongation and high concentration of CO_2_ (40 mM) available in developing oilseeds (Goffman *et al.*, 2004). Similarly, reversibility of IDH was observed in the TCA cycle of soybean embryos, even when the concentration of CO_2_ was lower (Allen *et al.*, 2009).

**Table 5.** MID and average labeling abundance (%) per carbon of free amino acids

Amino acids	Mass isotopomers	Average labeling (% ^13^C abundance)	
		[^13^C]GLC	[^13^C]Gln
Arg	M+0	64.38±0.63	6.25±0.49
	M+1	24.60±0.99	2.92±0.39
	M+2	5.90±0.50	2.25±0.66
	M+3	2.88±0.55	6.73±0.35
	M+4	1.44±0.47	4.80±0.29
	M+5	0.69±0.32	59.45±1.85
	M+6	0.11±0.27	18.24±1.97
Asn	M+0	57.28±0.49	30.86±0.90
	M+1	12.94±1.21	10.26±1.93
	M+2	15.90±1.26	16.68±1.71
	M+3	10.27±1.74	12.74±1.87
	M+4	3.74±0.69	30.01±1.04
Glu*	M+0	46.92±0.50	12.67±0.81
	M+1	11.64±1.25	3.18±0.54
	M+2	21.08±1.45	4.70±0.83
	M+3	10.66±3.19	12.09±0.92
	M+4	7.60±0.43	5.48±0.59
	M+5	2.11±0.59	61.88±2.23
Gln	M+0	89.70±0.61	4.24±0.73
	M+1	3.72±2.13	0.31±0.32
	M+2	4.87±3.49	4.23±1.52
	M+3	0.76±0.86	0.05±0.25
	M+4	0.56±0.61	1.81±0.65
	M+5	0.40±0.31	90.81±2.03
Gly	M+0	60.63±2.35	79.20±4.45
	M+1	26.80±1.43	7.15±3.41
	M+2	12.80±1.37	11.05±2.41
C1-Gly	M+0	81.13±1.86	81.61±1.32
	M+1	18.87±1.86	18.39±1.32
His	M+0	6.82±0.92	90.66±0.47
	M+1	13.23±0.95	8.22±0.66
	M+2	21.25±0.94	0.08±0.63
	M+3	21.84±1.01	0.31±0.59
	M+4	15.85±1.21	0.25±0.33
	M+5	14.83±1.18	0.31±0.63
	M+6	6.10±0.87	0.15±0.38
C1-His	M+0	53.12±0.44	98.03±0.80
	M+1	46.88±0.44	1.97±0.80
Ile	M+0	33.36±3.69	34.92±3.53
	M+1	10.26±1.30	11.22±2.07
	M+2	30.39±2.06	7.01±1.99
	M+3	11.16±3.29	9.00±3.93
	M+4	9.60±2.56	34.13±2.76
	M+5	4.17±1.67	2.11±2.19
	M+6	1.09±0.69	1.64±0.50
C1-Ile	M+0	84.86±2.41	57.80±1.67
	M+1	15.14±2.41	42.21±1.67
Met	M+0	28.57±2.62	37.05±0.73
	M+1	32.62±2.02	10.90±0.87
	M+2	15.34±1.01	7.75±1.07
	M+3	14.51±1.55	20.44±1.22
	M+4	7.70±0.35	24.98±1.52
	M+5	1.75±0.78	0.09±0.47
C1-Met	M+0	75.44±1.48	53.35±0.99
	M+1	24.56±1.48	46.65±0.99
Phe	M+0	9.15±0.81	88.70±0.48
	M+1	5.59±0.29	10.22±0.38
	M+2	15.22±0.68	0.58±0.36
	M+3	15.63±0.59	0.41±0.39
	M+4	16.76±0.60	0.05±0.29
	M+5	15.75±0.33	0.00±0.26
	M+6	10.90±0.62	0.00±0.26
	M+7	6.17±0.27	0.01±0.25
	M+8	3.73±0.36	0.03±0.26
	M+9	1.13±0.27	0.00±0.25
C1-Phe	M+0	71.46±0.52	97.90±0.37
	M+1	28.54±0.52	2.11±0.37
Ser*	M+0	32.66±2.55	81.28±2.31
	M+1	18.70±4.96	10.75±5.03
	M+2	29.99±3.72	1.43±5.82
	M+3	18.66±8.72	6.55±3.85
Val	M+0	17.35±0.51	79.76±1.13
	M+1	7.55±0.36	7.29±0.33
	M+2	32.43±0.63	5.44±0.78
	M+3	16.37±0.39	6.87±0.64
	M+4	16.22±0.56	0.32±0.30
	M+5	10.25±0.38	0.33±0.30
C1-Val	M+0	70.32±0.36	89.95±0.78
	M+1	29.68±0.36	10.05±0.78

Each mass isotopomer was detected and measured through LC-MS/MS in a positive ion mode. MID and the average labeling abundance (%) in each carbon were determined for free amino acids from [^13^C]GLC and [^13^C]Gln. The values listed are the average of three biological replicates ±SD. Labeling of carboxyl fragments resulting from LC-MS/MS analysis is included as ‘C1’ of the corresponding amino acid. An asterisk (*) indicates an amino acid whose MID values were corrected using the dilution factor from the 20% labeling experiment (Supplementary Table S6).

**Fig. 6.**
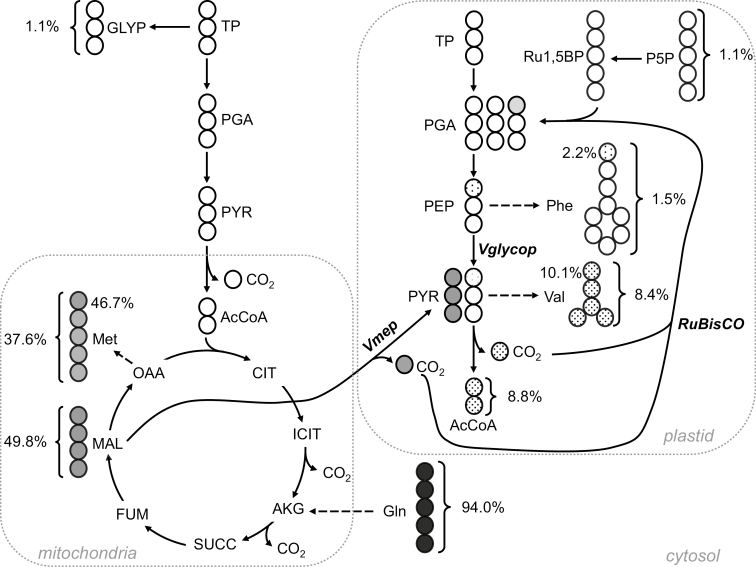
Simplified pathways illustrating carbon refixation by Rubisco in the plastid after labeling pennycress embryos with [U-^13^C_5_]glutamine. Values next to brackets report the average labeling per carbon (%) of the molecule, whereas values next to carboxylic groups are the ^13^C enrichment (%) for the C1 of the molecules determined by LC-MS/MS. Gray and white spheres represent labeled and unlabeled carbon atoms, respectively. The shades of gray are proportional to the labeling. The carboxylic groups of methionine, valine, and phenylalanine are derived from those of malate, pyruvate, and phophoenolpyruvate, respectively. Abbreviations (in alphabetical order): AcCoA, acetyl-CoA; AKG, α-ketoglutarate; CIT, citrate; FUM, fumarate; GLYP, glycerol phosphate; ICIT, isocitrate; MAL, malate; OAA, oxaloacetate; PGA, phosphoglycerate; P5P, pentose 5-phosphates; PYR, pyruvate; Ru1,5BP, ribulose 1,5-bisphosphate; SUCC, succinate; TP, triose phophates; *Vglycop*, portion of pyruvate produced from the plastidic glycolysis; *Vmep*, portion of pyruvate produced by the plastidic NADP-dependent malic enzyme.

Glycine had a labeling enrichment of 14.62±3.75% (Table 5), which is significantly more abundant than the labeling enrichment measured from its usual direct precursors in glycolysis, phosphoglycerate (PGA). This high labeling abundance in glycine underlines the activity of threonine aldolase (EC 4.1.2.5), which synthesizes glycine from threonine under non-photorespiratory conditions (Jander *et al.*, 2004; Joshi *et al.*, 2006; Jander and Joshi, 2010). Furthermore, the average labeling per carbon for valine (Table 5; Fig. 6), which is synthesized from pyruvate in the plastid, was found to be 8.34±0.55%. This value is significantly higher than the labeling enrichment of PEP (1.39±0.33%) and other upstream intermediates of glycolysis. Thus, this result reflects the operation of NADP-dependent malic enzyme (NADP-ME), which produces pyruvate from the decarboxylation of malate transported in the plastid.

From a study of ^13^C-labeled monounsaturated FA in rapeseed embryos, Rubisco was found to be refixing internally released ^13^CO_2_ without the Calvin cycle to increase the efficiency with which carbon is utilized for oil synthesis (Schwender *et al.*, 2004). In this rapeseed study, the refixation of ^13^CO_2_ by Rubisco was elucidated by the labeling pattern of tyrosine, valine, and phenylalanine that were synthesized from PEP and pyruvate (PYR) in the plastid. In the presence of ^13^CO_2_, rapeseed embryos were refixing ^13^CO_2_, resulting in the labeling of the C1 of PGA, the product of Rubisco. The labeled C1 of PGA became the C1 of PEP and PYR, and then was released as CO_2_ by pyruvate dehydrogenase. The C1 of PEP and PYR were also reflected by carboxylic groups of phenylalanine, tyrosine, and valine. This finding led us to consider labeling abundances of ^13^C1 fragments from these amino acids (Fig. 6) as indicators of the C1 of PEP and PYR in developing pennycress embryos. The percentage abundances of ^13^C1 from valine and phenylalanine were determined to be at 10.05±0.53% and 2.10±0.12%, respectively (Fig. 6). These values are significantly higher than the natural abundance of ^13^C (1.07%). Thus, it indicates that Rubisco recaptures internally released CO_2_ in pennycress embryos, producing PYR and PEP labeled on their C1, which in turn were reflected by the labeled carboxylic groups of these amino acids. Additionally, NADP-ME releases CO_2_ labeled at 46.7% as a result of decarboxylation of malate into pyruvate, contributing to the total labeled CO_2_ in the plastid that is available for Rubisco (Fig. 6). First, the relative contributions of plastidic glycolysis and NADP-ME to the pyruvate pool can be calculated according to Equation 9:

$$\begin{array}{l} \begin{array}{ll} & Vglycop+Vmep=1\\ & Vglycop\;\times \;C1\left(Phe\right)+Vmep\times C1\left(Met\right)\\ & \;=\left(Vglycop+Vmep\right)\times C1(Val) \end{array} \end{array}$$ (9)

where *Vglycop* and *Vmep* are the rates of plastidic glycolysis and NADP-ME, respectively. C1(Phe), C1(Val), and C1(Met) correspond to the labeling abundances of C1 fragments of phenylalanine, valine, and methionine, respectively (Table 5). As a result, *Vglycop* and *Vmep* were estimated to be 0.83 and 0.17, respectively, meaning that 17% of the PYR in the plastid is produced by the plastidic NADP-ME. Secondly, the labeling enrichment of plastidic CO_2_, denoted as *F*(CO_2_), can be estimated using Equation 10.

$$\begin{array}{ll} F\left(CO_{2}\right)= & \;\left(Vglycop+Vmep\right)\times C1\left(Val\right)\\ & +Vmep\times C1\left(Met\right) \end{array}$$ (10)

Given these measurements, *F*(CO_2_) was estimated to be 18% in the plastid. Finally, the relative contributions of Rubisco to PGA synthesis in the plastid was calculated using Equation 11, as previously described (Schwender *et al.*, 2004):

$$\begin{array}{l} \frac{2\;\;\;F\left(C1\;\;\;of\;\;\;PGA\right)}{F\left(CO_{2}\right)}=PGA\;\;\;from\;\;\;Rubisco \end{array}$$ (11)

where *F*(C1 of PGA) and *F*(CO_2_) are the labeling enrichments of the C1 of PGA and CO_2_, respectively. For *F*(C1 of PGA), the labeling abundance of the C1 of Phe (2.10%) was used. Thus, the overall contribution of Rubisco to PGA synthesis was ~25% in developing pennycress embryos.

In fact, Rubisco was previously shown to be responsible for a high CCE of 86% in rapeseed and 82% in soybean embryos by refixing internally released CO_2_ without the Calvin cycle (Schwender *et al.*, 2004; Allen *et al.*, 2009). Labeling pennycress embryos with [^13^C]glutamine showed that Rubisco was responsible for assimilating CO_2_ released from plastidic pyruvate dehydrogenase and malic enzyme, and for synthesizing 25% of the total PGA produced in the plastid (Fig. 6). In comparison, Rubisco was found to be producing 39% and 11% of PGA in rapeseed and soybean embryos, respectively (Schwender *et al.*, 2004; Allen *et al.*, 2009).

In conclusion, pennycress embryos convert carbon into storage oil with a high efficiency using non-conventional pathways that enable CO_2_ refixation: (i) the isocitrate dehydrogenase, regarded as catalyzing a thermodynamically irreversible decarboxylation, is operating reversibly in developing pennycress embryos, fixing CO_2_ to produce isocitrate and sustain fatty acid elongation; and (ii) Rubisco is functionally active in pennycress embryos, recapturing internally released CO_2_ and providing carbon skeletons for *de novo* oil synthesis.

## Supplementary data

Supplementary data are available at *JXB* online.

Fig. S1. The fatty acid composition of the oil from cultured and *in planta* embryos.

Table S1. Abundance of amino acids in storage proteins.

Table S2. Concentrations of sugars and sugar alcohols in pennycress endosperm.

Table S3. Concentrations of amino acids in pennycress endosperm.

Table S4. Concentrations of hormones in pennycress endosperm.

Table S5. Observed and expected biomass production and CO_2_ released by pennycress embryos in culture.

Table S6. The labeling abundance (%) per carbon of each metabolite from 20% [^13^C]glucose and 20% [^13^C]glutamine experiment.

eraa060_suppl_Supplementary_Material

## Abbreviations

CCE: carbon conversion efficiency
DAP: days after pollination
FA: fatty acid
FABA: fatty acid butyl amide
FAME: fatty acid methyl ester
MID: mass isotopomer distribution
MRM: multiple reaction monitoring
OPPP: oxidative pentose-phosphate pathway
TCA: tricarboxylic acid
