# Supplementary material for: Non-conventional pathways enable pennycress (Thlaspi arvense L.) embryos to achieve high efficiency of oil biosynthesis
Source: J Exp Bot. 2020 Feb 1;71(10):3037–51. doi: 10.1093/jxb/eraa060 (PMC7260723; doi:10.1093/jxb/eraa060)
Supplement: eraa060_suppl_Supplementary_Material [file eraa060_suppl_supplementary_material.pdf]

SUPPLEMENTARY DATA

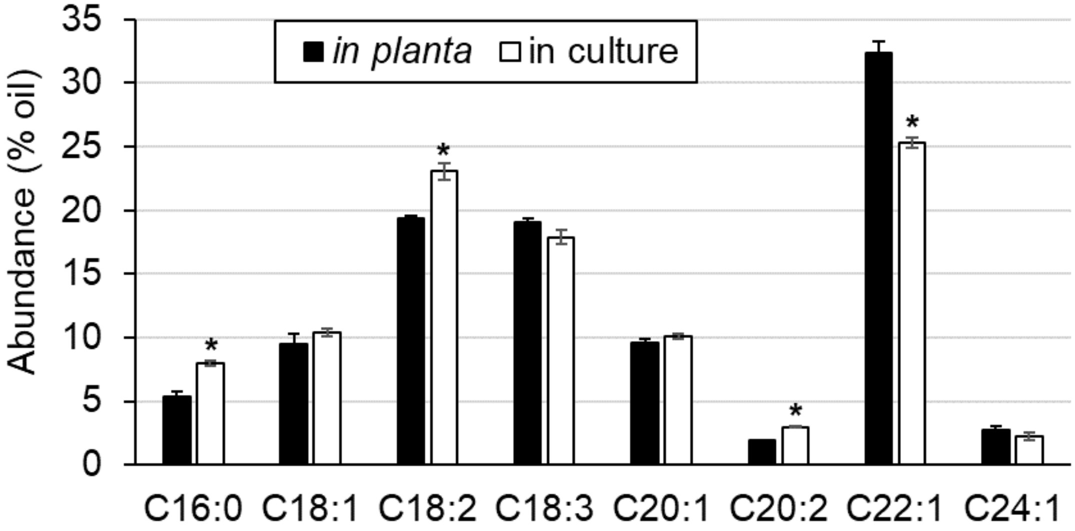

Supplementary Figure S1: The fatty acid composition of the oil from cultured and in planta embryos.

The percentage abundance and the error bars of each fatty acid species is an average and standard deviation of three biological replicates (n=3). C16:0, C18:1, C18:2, C18:3, C20:1, C20:2, C22:1 and C24:1 represent palmitic, oleic, linoleic, α-linolenic, gondoic, eicosadienoic, erucic and nervonic acids, respectively. An asterisk (*) indicates a statistically significant difference ($p < 0.05$).

Supplementary Table S1. Abundance of amino acids in storage proteins. Proteinogenic amino acids were quantified from in culture embryos through LC-MS/MS using an external standard containing a mixture of amino acids and the ^{13}C-glycine as an internal standard. The values are the average abundances ± standard deviation from three biological replicates.

Amino acids	Abundance (%)
Ala	6.28 ± 0.30
Arg	4.47 ± 0.06
Asp	9.41 ± 0.19
Glu	27.16 ± 0.72
Gly	5.68 ± 0.13
His	2.20 ± 0.02
Ile	8.13 ± 0.20
Leu	7.84 ± 0.20
Lys	5.69 ± 0.10
Met	0.41 ± 0.25
Phe	4.57 ± 0.06
Pro	0.12 ± 0.01
Ser	4.23 ± 0.12
Thr	3.65 ± 0.06
Tyr	3.49 ± 0.10
Val	6.86 ± 0.05

Supplementary Table S2. Concentrations of sugars and sugar alcohols in pennycress endosperm. Sugars and sugar alcohols were quantified using MRM scan survey as indicated in the Material and Methods section. The concentrations of metabolites are the average ± standard deviation of three biological replicates (n=3). "<1" represents metabolites with average concentrations lower than 1 mM. "n.d" corresponds to "not detected".

Sugars and sugar alcohols	Concentration (mM)
Glucose	49.34 ± 5.06
Fructose	34.92 ± 4.86
Sucrose	6.59 ± 2.29
Sorbitol	1.88 ± 0.80
Maltose	<1
Galactose	<1
Arabinose	<1
Mannitol	<1
Erythritol/Threitol	<1
Glycerol	<1
Pentitol	<1
Inositol	<1
Trehalose	n.d
Mannose	<1
Ribose	<1
Xylose	<1
Mannitol	<1
Galactitol	<1

Supplementary Table S3. Concentrations of amino acids in pennycress endosperm. Amino acids were quantified using MRM scan survey as indicated in the Material and Methods section. The concentrations of metabolites are the average ± standard deviation of three biological replicates (n=3). "<1" represents metabolites with average concentrations lower than 1 mM. "n.d" refers to "not detected".

Amino acids	Concentration (mM)
Ala	3.45 ± 0.17
Arg	1.18 ± 0.04
Asn	2.22 ± 0.46
Asp	1.77 ± 0.09
Citrulline	0.03 ± 0.01
Cys	n.d
GABA	1.69 ± 0.26
Gln	40.42 ± 12.82
Glu	2.30 ± 0.31
Gly	1.12 ± 0.07
His	0.90 ± 0.02
Hydroxyproline	0.37 ± 0.11
Ile	2.27 ± 0.21
Leu	0.72 ± 0.01
Lys	0.53 ± 0.03
Met	0.37 ± 0.01
Ornithine	0.06 ± 0.01
Phe	0.35 ± 0.03
Pro	3.28 ± 0.18
Ser	2.57 ± 0.16
Thr	4.20 ± 0.62
Trp	0.36 ± 0.09
Tyr	0.26 ± 0.02
Val	3.48 ± 0.18

Supplementary Table S4. Concentrations of hormones in pennycress endosperm. Hormones were extracted and quantified through LC-MS/MS as described in the Methods section. The quantities of metabolites reported here are the average ± standard deviation of three biological replicates (n=3). "n.d" corresponds to "not detected".

Hormones	Concentration (μM)
Abscisic acid	0.37 ± 0.01
Salicylic acid	0.28 ± 0.05
Gibberellic acid	n.d

Supplementary Table S5. Measured and expected biomass production and CO_2 released by pennycress embryos in culture. The values are average of three biological replicates (n=3).

Carbon sinks	Carbon observed (%)	Carbon expected by conventional pathways (%)
Oil	30.7	< 24.8
Protein, starch, and cell wall	62.8	62.8
CO_2 released	6.6	>12.4
Ratio of carbon in oil to CO_2	4.7	<2.0

Supplementary Table S6. **Labeling abundance (%) per carbon of each metabolite from 20 % ^{13}C-glucose and 20 % ^{13}C-glutamine experiments.** Each percentage value is the average of two replicates (n=2). Metabolites with labeling enrichment outside the range of 20 ± 3 % are in bold. Metabolites with labeling abundance higher than 23 % were excluded from data analysis due to contaminations, whereas those with less than 17 % enrichment were corrected according to the percentage shown in this table.

Class of metabolites	Names	Labeling abundance (%) per carbon
Amino acids	**Ala**	**24.49**
	Arg	19.76
	Asn	20.58
	**Glu**	**10.67**
	Gln	18.74
	Gly	17.36
	His	21.22
	Ile	22.17
	Met	18.11
	Phe	19.92
	**Ser**	**17.00**
	**Thr**	**23.06**
	Val	21.72
Organic acids	Citrate (CIT)	18.45
	Isocitrate (ICIT)	19.61
	Malate (MAL)	20.57
	**Succinate (SUCC)**	**13.78**
	Fumarate (FUM)	18.75
Phosphorylated compounds	Phosphoglycerate (PGA)	21.69
	6-phosphogluconate (6PG)	21.99
	Fructose 1,6-bisphosphate (F1,6BP)	21.70
	Glycerol phosphate (GLYP)	22.59
	Pentose 5-phosphateS (P5P)	21.20
	Phosphoenolpyruvate (PEP)	21.98
	Sedoheptulose 7-phosphate (S7P)	21.60
Compartimentalized metabolites	Plastidic acetyl-CoA unit (AcCoAp)	19.78
	**Cytosolic acetyl-CoA unit** (AcCoAc)	**15.57**
	Starch glucosyl unit (H6Pp)	19.58
	Sucrose glucosyl unit (G6Pc)	21.08
	Sucrose fructosyl unit (F6Pc)	20.87
	Glucose (GLCc)	22.60
